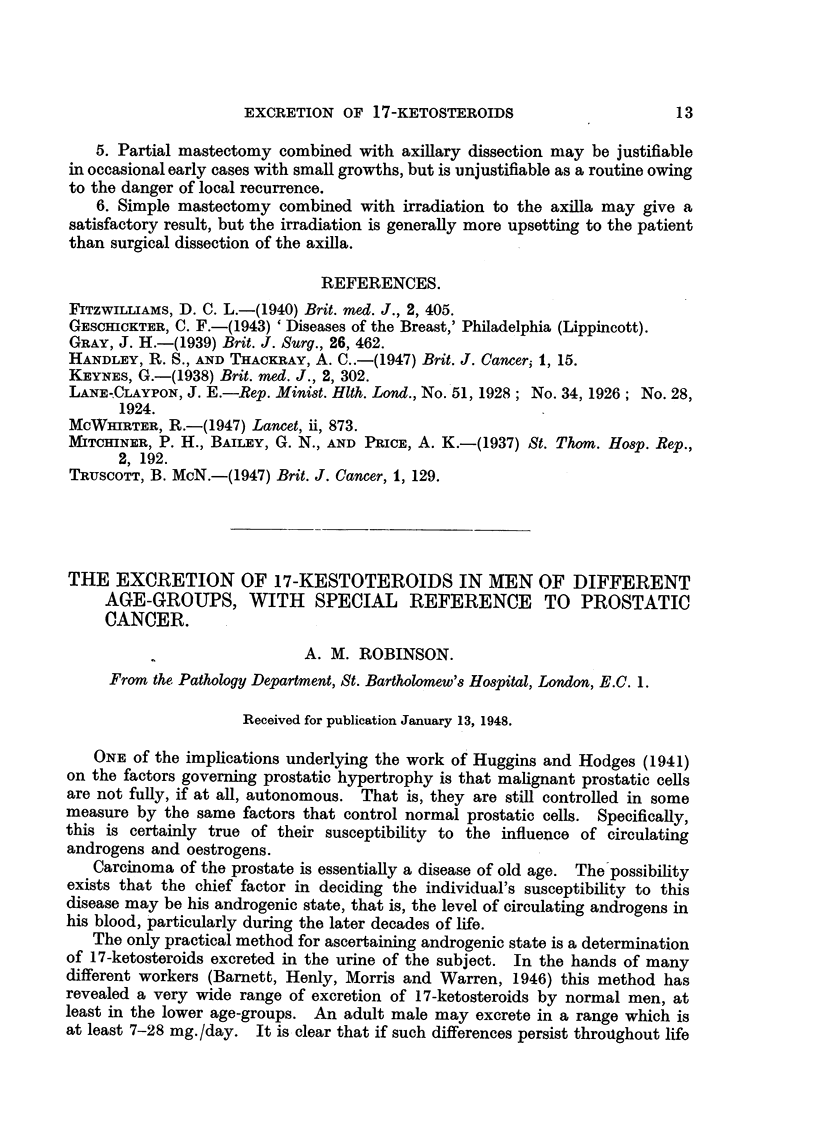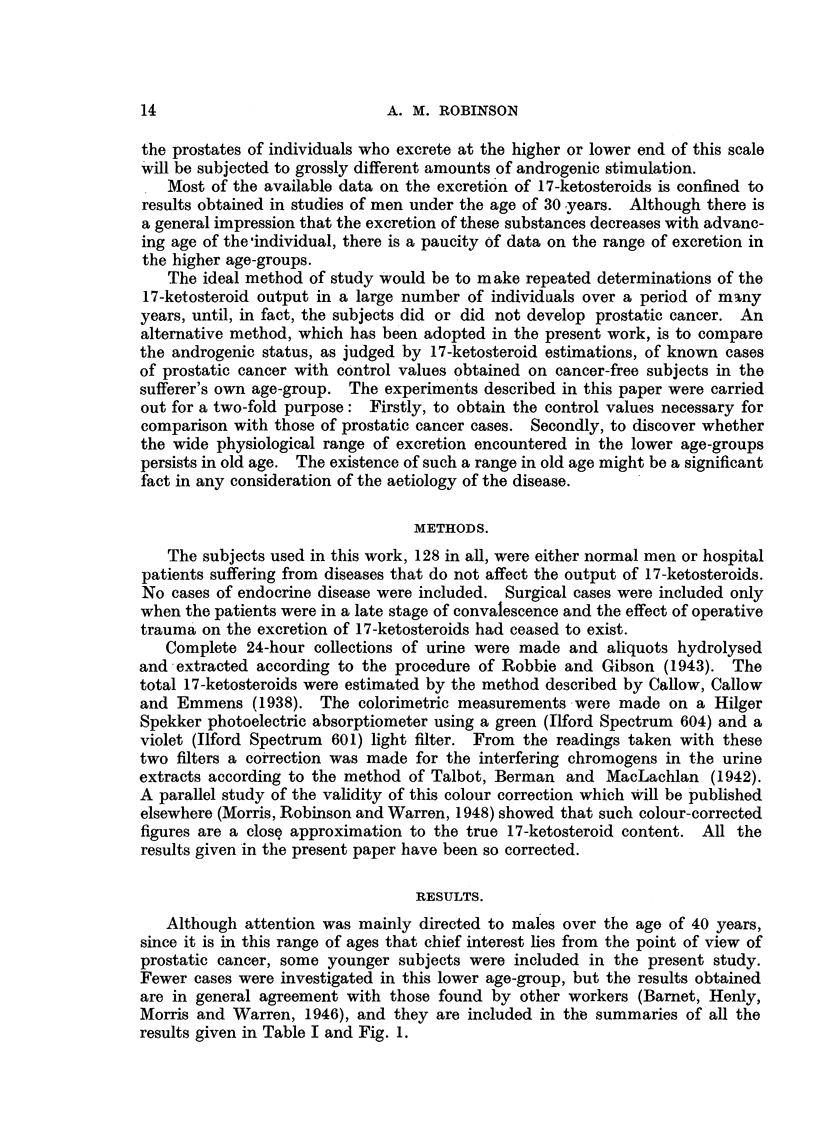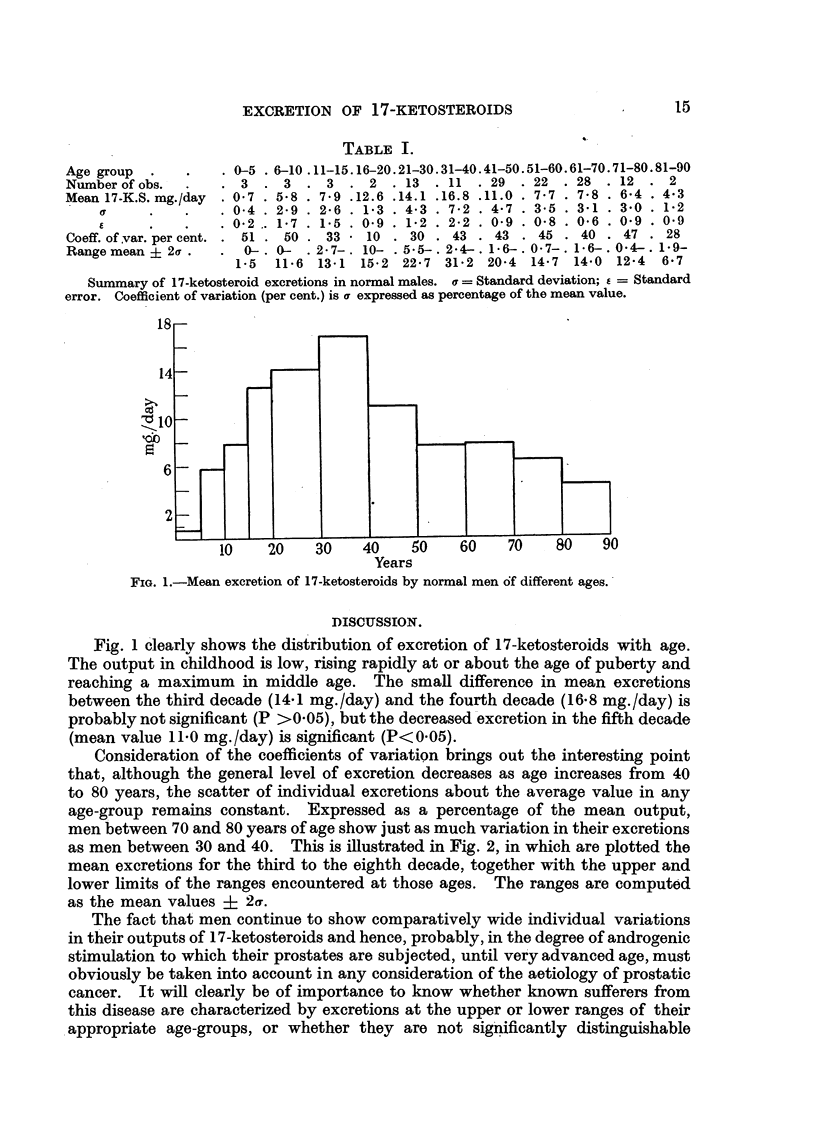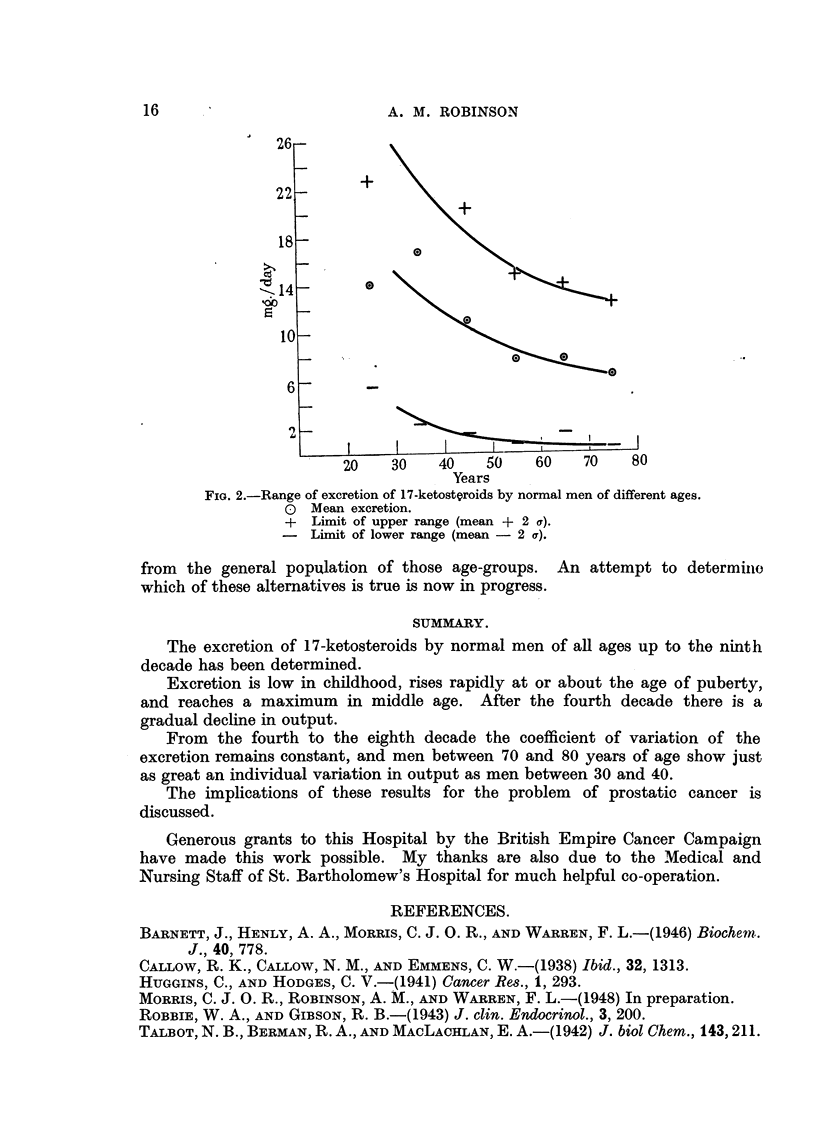# The Excretion of 17-Kestoteroids in Men of Different Age-Groups, with Special Reference to Prostatic cancer

**DOI:** 10.1038/bjc.1948.3

**Published:** 1948-03

**Authors:** A. M. Robinson


### THE EXCRETION OF 17-KESTOTEROIDS IN MEN OF DIFFERENT AGE-GROUPS, WITH SPECIAL REFERENCE TO PROSTATIC CANCER.

A. M. ROBINSON.

*From the Pathology Department, St. Bartholomew's Hospital, London, E.C.* 1.

Received for publication January 13, 1948.

ONE of the implications underlying the work of Huggins and Hodges (1941) on the factors governing prostatic hypertrophy is that malignant prostatic cells are not fully, if at all, autonomous. That is, they are still controlled in some measure by the same factors that control normal prostatic cells. Specifically, this is certainly true of their susceptibility to the influence of circulating androgens and oestrogens.

Carcinoma of the prostate is essentially a disease of old age. The possibility exists that the chief factor in deciding the individual's susceptibility to this disease may be his androgenic state, that is, the level of circulating androgens in his blood, particularly during the later decades of life.

The only practical method for ascertaining androgenic state is a determination of 17-ketosteroids excreted in the urine of the subject. In the hands of many different workers (Barnett, Henly, Morris and Warren, 1946) this method has revealed a very wide range of excretion of 17-ketosteroids by normal men, at least in the lower age-groups. An adult male may excrete in a range which is at least 7–28 mg./day. It is clear that if such differences persist throughout life the prostates of individuals who excrete at the higher or lower end of this scale will be subjected to grossly different amounts of androgenic stimulation.

Most of the available data on the excretion of 17-ketosteroids is confined to results obtained in studies of men under the age of 30 years. Although there is a general impression that the excretion of these substances decreases with advancing age of the individual, there is a paucity of data on the range of excretion in the higher age-groups.

The ideal method of study would be to make repeated determinations of the 17-ketosteroid output in a large number of individuals over a period of many years, until, in fact, the subjects did or did not develop prostatic cancer. An alternative method, which has been adopted in the present work, is to compare the androgenic status, as judged by 17-ketosteroid estimations, of known cases of prostatic cancer with control values obtained on cancer-free subjects in the sufferer's own age-group. The experiments described in this paper were carried out for a two-fold purpose: Firstly, to obtain the control values necessary for comparison with those of prostatic cancer cases. Secondly, to discover whether the wide physiological range of excretion encountered in the lower age-groups persists in old age. The existence of such a range in old age might be a significant fact in any consideration of the aetiology of the disease.

### METHODS.

The subjects used in this work, 128 in all, were either normal men or hospital patients suffering from diseases that do not affect the output of 17-ketosteroids. No cases of endocrine disease were included. Surgical cases were included only when the patients were in a late stage of convalescence and the effect of operative trauma on the excretion of 17-ketosteroids had ceased to exist.

Complete 24-hour collections of urine were made and aliquots hydrolysed and extracted according to the procedure of Robbie and Gibson (1943). The total 17-ketosteroids were estimated by the method described by Callow, Callow and Emmens (1938). The colorimetric measurements were made on a Hilger Spekker photoelectric absorptiometer using a green (Ilford Spectrum 604) and a violet (Ilford Spectrum 601) light filter. From the readings taken with these two filters a correction was made for the interfering chromogens in the urine extracts according to the method of Talbot, Berman and MacLachlan (1942). A parallel study of the validity of this colour correction which will be published elsewhere (Morris, Robinson and Warren, 1948) showed that such colour-corrected figures are a close approximation to the true 17-ketosteroid content. All the results given in the present paper have been so corrected.

### RESULTS.

Although attention was mainly directed to males over the age of 40 years, since it is in this range of ages that chief interest lies from the point of view of prostatic cancer, some younger subjects were included in the present study. Fewer cases were investigated in this lower age-group, but the results obtained are in general agreement with those found by other workers (Barnet, Henly, Morris and Warren, 1946), and they are included in the summaries of all the results given in Table I and Fig. 1.

TABLE I.

| Age group | 0–5 | 6–10 | 11–15 | 16–20 | 21–30 | 31–40 | 41–50 | 51–60 | 61–70 | 71–80 | 81–90 |
|---|---|---|---|---|---|---|---|---|---|---|---|
| Number of obs. | 3 | 3 | 3 | 2 | 13 | 11 | 29 | 22 | 28 | 12 | 2 |
| Mean 17-K.S. mg./day | 0·7 | 5·8 | 7·9 | 12.6 | 14.1 | 16.8 | 11.0 | 7·7 | 7·8 | 6·4 | 4·3 |
| $\sigma$ | 0·4 | 2·9 | 2·6 | 1·3 | 4·3 | 7·2 | 4·7 | 3·5 | 3·1 | 3·0 | 1·2 |
| $\epsilon$ | 0·2 | 1·7 | 1·5 | 0·9 | 1·2 | 2·2 | 0·9 | 0·8 | 0·6 | 0·9 | 0·9 |
| Coeff. of var. per cent. | 51 | 50 | 33 | 10 | 30 | 43 | 43 | 45 | 40 | 47 | 28 |
| Range mean $\pm$ $2\sigma$ | 0–1·5 | 0–11·6 | 2·7–13·1 | 10–15·2 | 5·5–22·7 | 2·4–31·2 | 1·6–20·4 | 0·7–14·7 | 1·6–14·0 | 0·4–12·4 | 1·9–6·7 |

Summary of 17-ketosteroid excretions in normal males. $\sigma$ = Standard deviation; $\epsilon$ = Standard error. Coefficient of variation (per cent.) is $\sigma$ expressed as percentage of the mean value.

FIG. 1.—Mean excretion of 17-ketosteroids by normal men of different ages.

### DISCUSSION.

Fig. 1 clearly shows the distribution of excretion of 17-ketosteroids with age. The output in childhood is low, rising rapidly at or about the age of puberty and reaching a maximum in middle age. The small difference in mean excretions between the third decade (14·1 mg./day) and the fourth decade (16·8 mg./day) is probably not significant ($P > 0·05$), but the decreased excretion in the fifth decade (mean value 11·0 mg./day) is significant ($P < 0·05$).

Consideration of the coefficients of variation brings out the interesting point that, although the general level of excretion decreases as age increases from 40 to 80 years, the scatter of individual excretions about the average value in any age-group remains constant. Expressed as a percentage of the mean output, men between 70 and 80 years of age show just as much variation in their excretions as men between 30 and 40. This is illustrated in Fig. 2, in which are plotted the mean excretions for the third to the eighth decade, together with the upper and lower limits of the ranges encountered at those ages. The ranges are computed as the mean values $\pm$ $2\sigma$.

The fact that men continue to show comparatively wide individual variations in their outputs of 17-ketosteroids and hence, probably, in the degree of androgenic stimulation to which their prostates are subjected, until very advanced age, must obviously be taken into account in any consideration of the aetiology of prostatic cancer. It will clearly be of importance to know whether known sufferers from this disease are characterized by excretions at the upper or lower ranges of their appropriate age-groups, or whether they are not significantly distinguishable

FIG. 2.—Range of excretion of 17-ketosteroids by normal men of different ages.
⊙ Mean excretion.
+ Limit of upper range (mean + 2 σ).
— Limit of lower range (mean — 2 σ).

from the general population of those age-groups. An attempt to determine which of these alternatives is true is now in progress.

SUMMARY.

The excretion of 17-ketosteroids by normal men of all ages up to the ninth decade has been determined.

Excretion is low in childhood, rises rapidly at or about the age of puberty, and reaches a maximum in middle age. After the fourth decade there is a gradual decline in output.

From the fourth to the eighth decade the coefficient of variation of the excretion remains constant, and men between 70 and 80 years of age show just as great an individual variation in output as men between 30 and 40.

The implications of these results for the problem of prostatic cancer is discussed.

Generous grants to this Hospital by the British Empire Cancer Campaign have made this work possible. My thanks are also due to the Medical and Nursing Staff of St. Bartholomew's Hospital for much helpful co-operation.

REFERENCES.

BARNETT, J., HENLY, A. A., MORRIS, C. J. O. R., AND WARREN, F. L.—(1946) *Biochem. J.*, **40**, 778.

CALLOW, R. K., CALLOW, N. M., AND EMMENS, C. W.—(1938) *Ibid.*, **32**, 1313.

HUGGINS, C., AND HODGES, C. V.—(1941) *Cancer Res.*, **1**, 293.

MORRIS, C. J. O. R., ROBINSON, A. M., AND WARREN, F. L.—(1948) In preparation.

ROBBIE, W. A., AND GIBSON, R. B.—(1943) *J. clin. Endocrinol.*, **3**, 200.

TALBOT, N. B., BERMAN, R. A., AND MACLACHLAN, E. A.—(1942) *J. biol Chem.*, **143**, 211.